# Media consumption and mental health during COVID-19 lockdown: a UK cross-sectional study across England, Wales, Scotland and Northern Ireland

**DOI:** 10.1007/s10389-021-01506-0

**Published:** 2021-03-20

**Authors:** Ruth D Neill, Carolyn Blair, Paul Best, Emily McGlinchey, Cherie Armour

**Affiliations:** 1 School of Social Sciences, Education and Social Work, Queen’s University Belfast, 6 College Park Ave, Belfast, Northern Ireland BT7 1PS UK; 2 Stress, Trauma, and Related Conditions (STARC) Research Lab, School of Psychology, Queen’s University Belfast, Malone Road, Belfast, Northern Ireland BT9 5BN UK

**Keywords:** COVID-19, Mental health, Media consumption, Anxiety, Depression

## Abstract

**Aim:**

As individuals adjust to new ‘norms’ and ways of living during the COVID-19 lockdown, there is a continuing need for up-to-date information and guidance. Evidence suggests that frequent media exposure is related to a higher prevalence of mental health problems, especially anxiety and depression. The aim of this study was to determine whether COVID-19 related media consumption is associated with changes in mental health outcomes.

**Methods:**

This paper presents baseline data from the COVID-19 Psychological Wellbeing Study. The cross-sectional study data was collected using an online survey following the Generalised Anxiety Disorder scale (GAD-7) and the Patient Health Questionnaire (PHQ-9), with some other basic information collected. Logistic regression analysis was used to examine the influence of socio-demographic and media specific factors on anxiety and depression.

**Results:**

The study suggested that media usage is statistically significantly associated with anxiety and depression on the GAD-7 and PHQ-9 scales with excessive media exposure related to higher anxiety and depression scores.

**Conclusion:**

This study indicated that higher media consumption was associated with higher levels of anxiety and depression. Worldwide it should be acknowledged that excessive media consumption, particularly social media relating to COVID-19, can have an effect on mental health. However, as this was a cross-sectional study we cannot infer any directionality as we cannot infer cause and effect; therefore, future research involving longitudinal data collection and analyses of variables over time is warranted.

## Introduction

The outbreak of coronavirus disease 2019 (COVID-19) has created a global health crisis that has had a deep impact on the way we perceive our world and our everyday lives (Van Bavel et al. 2020). The rate of contagion and patterns of transmission has threatened our sense of agency. Furthermore, the safety measures put in place to contain the spread of the virus through social and physical distancing has limited our ability to find solace in the company of others. Within this context of physical threat, social and physical distancing, the use of media channels (including social media and traditional media) to acquire and exchange information has increased at a historic and unprecedented scale (Allington et al. 2020; Chao et al. 2020; Li et al. 2020).

People use social media to acquire and exchange various types of information at a historic and unprecedented scale (Li et al. 2020). Media usage can positively influence individuals by raising awareness, reporting important public health messages and promoting positive health behaviours such as social distancing, handwashing and good hygiene, which can help reduce disease transmission (Collinson et al. 2015; Sahni and Sharma 2020). However, in response to the epidemic, only the situational information is deemed valuable for the public and authorities. The official departments strive to improve the public’s awareness of prevention and intervention strategies by providing daily updates about surveillance and active cases on websites and social media (Bao et al. 2020). This method of sharing and validating information contrasts with methods more directly controlled by intermediaries (e.g. traditional media), who have specialised knowledge and specific responsibilities related to information verification and sharing (Eysenbach 2007). This information-sharing model has become a driving feature of how public information related to health and medicine is produced and disseminated.

Limaye et al. (2020) have reported that during the COVID-19 pandemic, individuals are increasingly turning to these media channels for guidance. Research suggests that these media sources are often behind an increase in stress, which contributes to major psychological problems associated with receiving conflicting messages related to COVID-19 (Garfin et al. 2020; Pfefferbaum and North 2020). These information-seeking behaviours through traditional and social media can lead to uncertainty, thus affecting one’s mental state and triggering anxiety and depression related symptoms (Chao et al. 2020; Ebrahim et al. 2020; Liu 2020; Moreno et al. 2020).

High COVID-19 related social media consumption may lead to (mis)information overload and consequently increase levels of anxiety and depression, despite efforts to seek reassurance, which in turn may impact mental health (Garfin et al. 2020; World Health Organization 2020). During the COVID-19 outbreak, Gao et al.’s (2020) findings show a high prevalence of mental health problems, especially depression and anxiety, which are positively associated with frequent social media exposure. However, despite initial research, the impact of increased social and traditional media usage on mental health and mental-being through the COVID-19 pandemic is yet to be fully established. Therefore this study will examine the relationship between COVID-19 related media consumption (social media and traditional media), anxiety and depression at the beginning of lockdown in the United Kingdom (UK). This paper explores whether media consumption is a predictor of anxiety and depression, therefore extending the current literature by examining the impact of different media platforms.

## Methods

### Research design

The study conducted was based on cross-sectional data. The baseline data presented here is taken from the COVID-19 Psychological Wellbeing Study (Armour et al. 2020) a longitudinal, online survey of the general adult (18+) population of the UK. The study was designed to assess and monitor the psychosocial impact of the COVID-19 pandemic on UK residents. Baseline data collection began on 23rd March 2020 (commencement of the UK’s lockdown period) and closed on 24th April 2020.

### Recruitment and sampling

Participants were recruited via (1) a large-scale social media campaign and (2) using an online participant panel called Prolific. The survey was administered online through the survey data collection platform ‘Qualtrics’ after participants provided informed consent to participate in the study.

### Inclusion criteria

All participants were 18+ years or older, currently resident in the UK and able to read and write in English. Participants were removed from the study for not providing consent, failing to meet the inclusion criteria, (i.e. UK residents over the age of 18 years old), not completing any of the outcome measures or completing the survey in less than half the median completion time.

### Ethical approval

All participants received a participant information sheet outlining the purpose of the study, how information would be stored, what would happen to the information concerning onward publication of the data and the results, and the risks and benefits associated with participation. Ethical approval was provided by the faculty of Engineering and PhysicalSciences at Queen’s University Belfast (Reference:EPS 20_96) and also Glasgow Caledonian UniversityHealth and Life Sciences Ethics Committee, (HLS/PSWAHS/19/157).

### Measures

A number of standardised measures were included in the survey. However, for this paper’s purposes, the focus was mainly on variables relating to media consumption and mental health issues such as anxiety and depression (described below). We included specific socio-demographic data (age and gender) within our analysis.

#### Media/information consumption

Participants were asked three items in relation to the degree they consumed COVID-19 information via the media. They were asked how often they were watching, reading and hearing reports or updates about COVID-19 on either (1) social media or (2) traditional media. The response categories were (1) less than once a day, (2) 1–5 times a day, (3) 6–10 times a day, (4) 11–20 times a day, (5) 20–50 times a day and (6) more than 50 times a day. This was consistent for each of the three types of media queried. Media exposure variables were then recoded in low (0–5 times a day), moderate (6–20 times a day) or high (20 or more times a day) media consumption categories.

#### Generalised anxiety disorder

The Generalised Anxiety Disorder scale (GAD-7) (Spitzer et al. 2006) measures symptoms of generalised anxiety. The scale focuses on symptoms experienced over the past two weeks. The scale contains seven items, with each item ranging from 0 (not at all) to 3 (nearly every day). The psychometric properties of the GAD-7 are well acknowledged (Lee and Kim 2019; Rutter and Brown 2017; Spitzer et al. 2006). A score of 0–4 is considered to be normal levels of anxiety, 5–9 is considered mild, 10–14 is moderate while 15–21 is severe (Spitzer et al. 2006). Based on previous research, scores of 10 or above were considered to meet the clinical threshold criteria for GAD (Spitzer et al. 2006).

#### Major depressive disorder (MDD)

The Patient Health Questionnaire (PHQ-9) (Kroenke et al. 2001) was used to measure symptoms of major depressive disorder, over the past two weeks. The PHQ-9 contains nine items, based upon the DSM diagnostic criteria (American Psychiatric Association 2013). Each item is scored on a 4-point Likert scale, ranging from 0 to 4. The response categories were, not at all (0), several days (1), more than half the days (2) and nearly every day (3). All items are summed together to yield a total score, with a possible range of 0–27. A total score of 0–4 is considered none or mild, 5–9 is considered minimal, 10–14 is considered moderate, 15–19 is moderately severe and greater than 20 is severe. Based off previous research, scores of 10 or above were considered to meet the clinical threshold criteria for MDD (Kroenke et al. 2001). The PHQ-9 has shown utility across a number of clinical and non-clinical contexts (Levis et al. 2019; Umegaki and Todo 2017; Allgaier et al. 2012).

## Statistical analysis

Preliminary analyses using descriptive statistics and univariate analyses were conducted to examine the associations between participants’ socio-demographic, mental health, and media consumption variables. Independent t-tests and one-way ANOVAs were used to examine whether anxiety/depression levels differed based on media consumption levels (i.e. low usage, moderate usage, high usage). Factors found to be correlated to GAD and MDD scores were entered into a binary logistic regression model. Statistical tests were performed using SPSS version 25.0 for Windows (Armonk, MY, IBM Corp).

## Results

### Sample characteristics

A recruitment response target of participants of 2000 was the aim for the baseline survey, with the total response rate at 2501. After several exclusions were applied concerning data quality control (please see inclusion criteria), a final effective sample of 1989 participants from Great Britain and Northern Ireland aged between 18 and 87 (M = 37.11, SD = 12.86) were eligible to be included in baseline data. Overall, the sample was mostly female (70.4%) and white (92.7%). Overall, 30.3% of the sample met the threshold criteria for GAD, mean score on GAD-7 scale, 7.27 (SD = 6.02), while 34% met the criteria for MDD, mean score on PHQ-9 scale, 7.84 (SD = 6.48). The remaining sample characteristics are presented in Table 1.

**Table 1:** Social demographic, mental health variables, media consumption by GAD-7 and PHQ-9 scores

	N (%)	GAD-7 mean scores (SD)	PHQ-9 mean scores (SD)
**Overall**	1989 (100)	7.27 (6.01)	7.84 (6.48)
**Age**			
18–24	331 (16.6)	8.41 (6.49)	10.01 (7.03)
25–34	659 (33.1)	8.00 (6.05)	8.47 (6.45)
35–44	476 (23.9)	7.33 (5.81)	7.64 (6.45)
45–54	291 (14.6)	6.40 (5.65)	6.7 (5.92)
55+	232 (11.7)	4.48 (5.05)	4.69 (5.28)
**Gender**			
Female	1392 (70)	7.98 (6.07)	8.24 (6.43)
Male	582 (29.3)	5.46 (5.45)	6.70 (6.33)
**Country**			
Northern Ireland	470 (23.6)	7.73 (5.88)	7.65 (6.05)
England	747 (37.6)	7.02 (5.89)	8.01 (6.58)
Scotland	726 (36.5)	7.29 (6.22)	7.84 (6.66)
Wales	46 (2.3)	6.46 (6.15)	6.96 (6.23)
**Keyworker**			
Yes	743 (37.4)	7.33 (5.97)	7.64 (6.18)
No	1243 (62.6)	7.23 (6.05)	7.97 (6.65)
**Living alone**			
Yes	399 (20.1)	7.05 (5.97)	8.39 (6.81)
No	1585 (79.9)	7.32 (6.03)	7.70 (6.40)
**Meets clinical threshold for GAD**			
Yes	596 (30.3)	15.09 (3.68)	14.61 (5.81)
No	1374 (69.7)	3.88 (2.84)	4.90 (4.12)
**Meets clinical threshold for MDD**			
Yes	668 (34.0)	13.10 (5.23)	15.45 (4.50)
No	1287 (66.0)	4.28 (3.78)	3.91 (2.81)
**Media consumption** ***Traditional media*** *(*e.g. *newspapers, TV, radio)*			
Low	1522 (77.0)	6.91 (5.90)	7.45 (6.32)
Moderate	376 (19.0)	8.14 (6.04)	8.80 (6.66)
High	78 (3.9)	10.08 (7.08)	10.86 (7.37)
***Social media*** *(*e.g. *Facebook, twitter, Instagram)*			
Low	1143 (57.8)	6.15 (5.56)	6.78 (6.10)
Moderate	611 (30.9)	8.40 (6.11)	8.84 (6.56)
High	222 (11.2)	9.98 (6.57)	10.56 (6.97)

### Factors for increasing MDD and GAD scores

#### Univariate analysis

The results demonstrated that as media usage increased, so too did scores on the GAD-7 and MDD (Table 1). Both traditional and social media consumption variables were statistically significant and positively correlated with GAD-7 and PHQ-9 scales. GAD-7 and PHQ-9 scales were highly and positively correlated with each other (Table 2).

**Table 2:** Correlations for study variables

Variables	Social media usage	Traditional media usage	Living alone	Keyworker status	Gender	Age	GAD	MDD
Social media usage								
Traditional media usage	.302^**^							
Living alone	0.052*	.014						
Keyworker status	0.049*	.059**	.001					
Gender	.082^**^	−.077^**^	.003	1.00**				
Age	−.207^**^	.029	.143**	.030	.018			
GAD	.239^**^	.101^**^	−.018	.734	.192^**^	−.187^**^		
MDD	.223^**^	.100^**^	−0.43	.288	.109^**^	−.228^**^	.819^**^	

**. *p* < 0.01 level

*. *p* < 0.05 level

Results from an independent t-test demonstrated there was a significant difference in GAD scores for low social media consumption (M = 6.15, SD = 5.56) and high social media consumption (M = 9.98, SD = 6.57; t(284.18) = −8.12, *p* < 0.001). The magnitude of differences in the means (mean difference = −3.83, 95% CI = − 4.76 to −2.90) was large (Cohen’s d = 0.63). Similarly, there was a significant difference in GAD scores for low traditional media consumption (M = 6.91, SD = 5.90) and high traditional media consumption (M = 10.08, SD = 7.08); t(82.58) = −3.88, *p* = 0.00). The magnitude of differences in the means (mean difference = −3.17, 95% CI = − 4.79 to −1.54) was medium (Cohen’s d = 0.49). There was no significant difference in GAD scores (*p* > 0.05) for keyworker status (*p* = 0.73) or living status (*p* = 0.42).

Statistically significant differences were shown in MDD scores for low traditional media consumption (M = 7.45, SD = 6.32) and high traditional media consumption (M = 10.86, SD = 7.37; t(82.94) = −4.02, *p* < 0.001). The magnitude of differences in the means (mean difference = −3.41, 95% CI = − 5.10 to −1.72) was medium (Cohen’s d = 0.49). There was a significant difference in MDD scores for low social media consumption (M = 6.78, SD = 6.10) and high social media consumption (M = 10.56, SD = 6.97; t(285.64) = −7.49, *p* < 0.001). The magnitude of differences in the means (mean difference = −3.78, 95% CI = − 4.77 to −2.79) was large (Cohen’s d = 0.58). There was no significant difference in MDD scores (p > 0.05) for keyworker status (*p* = 0.30) or living status (*p* = 0.07).

#### Multivariate analysis

A statistically significant difference between all social media consumption categories (low, moderate and high) was determined by a one-way ANOVA (F(2, 1967) = 55.98, *p* < 0.001). A tukey post hoc test revealed that total GAD scores were statistically significantly higher with high social media consumption (M = 10.08, SD = 7.08) when compared to low (M = 6.91, SD = 5.90) or moderate (M = 8.14, SD = 6.04) social media consumption. Traditional media consumption categories also revealed a statistically significant difference with total GAD scores between which was determined by a one-way ANOVA (F(2, 1967) = 15.37, *p* < 0.001). A tukey post hoc test revealed that total GAD scores were statistically significantly higher with higher traditional media consumption (M = 10.08, SD = 7.08) when compared to low (M = 6.91, SD = 5.90) or moderate (8.14, 6.04) traditional media consumption.

MDD scores also demonstrated a statistically significant difference between all traditional media consumption categories (low, moderate and high), determined by a one-way ANOVA (F(2, 1962) = 43.45, p < 0.001). A tukey post hoc test revealed that total MDD scores were significantly higher with high traditional media consumption (M = 10.86, SD = 7.37) when compared to low (M = 7.45, SD = 6.32) or moderate (M = 8.80, SD = 6.66) traditional media consumption. Similarly, a statistically significant difference between all social media consumption groups (low, moderate and high) was determined by a one-way ANOVA (F(2, 1962) = 43.45, p < 0.001). A tukey post hoc test revealed that total MDD scores were significantly higher with high social media consumption (M = 10.56, SD = 6.97) when compared to low (M = 6.78, SD = 6.10) or moderate (M = 8.84, SD = 6.56) social media consumption.

A total of 4 of the included variables that may be influencing factors were significant (≤0.05) in the univariate analysis and were entered into a standard logistic regression. The living alone and keyworker status variables were excluded from the analysis as no significant differences were reported, *p* > 0.05. The final model for the various predictor variables is presented in Table 3.

**Table 3:** Logistic regression analysis of factors predicting meeting GAD or MDD clinical threshold criteria

GAD clinical threshold criteria				MDD clinical threshold		
	ß	OR (CI)	*p*	ß	OR (CI)	*p*
Gender	0.801	2.227 (1.752–2.830)	0.000***	0.389	1.476 (1.185–1.839)	0.001***
Age	−0.027	0.974 (0.965–0.982)	0.000***	−0.033	0.967 (0.959–0.975)	0.000***
Traditional media consumption	0.577	1.781 (1.283–2.472)	0.001***	0.674	1.963 (1.420–2.713)	0.000***
Social media consumption	0.423	1.527 (1.209–1.928)	0.000***	0.444	1.559(1.241–1.958)	0.000***
Constant	−.652	.521	0.001***	0.058	1.060	0.747

OR – odds ratio; CI – confidence intervals; ß – standardised regression coefficient; *p* – significance (***p < 0.001, **p < 0.01 level, *p < 0.05 level)

The logistic model was statistically significant, *x*^2^ (4) = 128.31, *p* < 0.001. The model explained 9.0% of the variance in meeting the clinical threshold for GAD and correctly classified 70.6% of cases. Females were 42.9 times more likely to meet the clinical threshold criteria for GAD than males. Increasing age was associated with an increased likelihood of meeting the clinical threshold criteria for GAD. Both high traditional and social media consumption variables were linked to increased GAD scores and an increased likelihood of meeting the clinical threshold criteria for GAD.

Similarly, the logistic model for MDD was statistically significant *x*^2^ (4) = 132.92, *p* < 0.001. The model explained 9.1% of the variance in meeting the clinical threshold for MDD and correctly classified 67.8% of cases. Females were 12.03 times more likely to meet the clinical threshold criteria for MDD than males. Lower age was associated with a lower likelihood of meeting the clinical threshold criteria for MDD. Both high traditional and social media consumption variables were linked to increased MDD scores and an increased likelihood of meeting the clinical threshold criteria for MDD.

## Discussion

The primary aim was to assess whether a relationship exists between COVID-19 media consumption and mental health issues such as anxiety and depression. These media consumption variables were positively associated with anxiety and depression. Previous research has highlighted that an outbreak of infectious disease such as COVID-19 can lead to increased anxiety and depression due to the negative impact information from the media can have on individuals’ mental health (Kumar and Somani 2020; Rajkumar 2020). Researchers have acknowledged that misunderstandings of COVID-19-related messages in the media can lead to this increase in anxiety and depression across the population (Bao et al. 2020; Chao et al. 2020; Garfin et al. 2020; Limaye et al. 2020; Torales et al. 2020). Evidence suggests that if individuals are repeatedly exposed through media consumption, then this is likely to provoke an increase in anxiety and depression (Ebrahim et al. 2020; Garfin et al. 2020; Huang and Zhao 2020; Lee 2020; Oh et al. 2020; Torales et al. 2020; Zheng et al. 2020).

Similarly, this study indicated that across both media consumption variables (social and traditional), higher exposure to media usage was correlated with higher scores in anxiety and depression. The data in this study suggested that daily media consumption of social media was higher than traditional media usage. Ebrahim et al. (2020) also reported that the frequency of media usage was significantly associated with GAD scores, which corresponds to this study’s findings. Similarly, Garcia-Priego et al. (2020) highlighted that internet usage in Mexico could have a significant association with anxiety and depression, while Chao et al. (2020) found that in China, new media was more significantly associated with mental health issues over traditional media usage. Additionally, Mowbray (2020) stated that since the COVID-19 outbreak, depression had increased by 7% with social media usage being one of the leading factors in this increase. A non-COVID-19 related study also demonstrated (Demirci et al. 2015) that depression and anxiety scores were higher in those with high social media consumption than those with lower media consumption. Additional evidence suggests that individuals with higher anxiety and depression levels are more likely to use social media at higher rates (Shensa et al. 2018; Vannucci et al. 2017). However, because this research is cross-sectional, we cannot assume that depression and anxiety levels are higher because of increased media usage as directionality of the association cannot be identified and other potential confounding factors were not examined.

Roy et al. (2020) found that in over 50% of study participants, anxiety increased due to constant discussion and COVID-19 media reports on electronic and print media. An online survey in 31 provinces in China found that social media exposure was positively associated with higher odds of anxiety and depression when compared to lower social media exposure (Gao et al. 2020). These results demonstrate that social media usage can be a statistically significant predictor of anxiety and depression, which is similar to the current findings. In addition, Ni et al. (2020) reported that excessive usage of >2 h on social media about COVID-19 was associated with anxiety and depression in adults. Furthermore, the authors reported that traditional media (news/television reports) was not associated with anxiety or depression. These findings confirm the regression in this study as traditional media usage was not found to be a predictor of anxiety or depression (*p* > 0.05).

Overall there is limited research, particularly within the entire United Kingdom, to examine the effects COVID-19 media consumption has on anxiety and depression. Although this study’s results do not provide sufficient evidence to recommend that anxiety and depression can be predicted by forms of media consumption, they confirm that a statistically significant relationship exists between media usage and mental health. Previous research has suggested that misinformation from media usage, particularly social media usage through Facebook or Twitter, can be more harmful to one’s mental health (Allington et al. 2020; Limaye et al. 2020; Ni et al. 2020). Whilst other evidence suggest social media may be beneficial in creating social support during this pandemic; however, this is only applicable for those who have access to these resources (Carlsen et al. 2020; Saud et al. 2020). Based off World Health Organization (2020) guidelines, while socialising over media platforms such as Facetime and Whatsapp can be useful for social support, it is important not to spend a significant portion of the day checking and scanning media platforms for information. Organisations such as the World Health Organization and government agencies need to educate the public on media consumption and which sources are reliable while acknowledging media usage should be limited during this pandemic.

As this is only phase 1 of the research, it will be essential to examine the changes in media consumption, anxiety and depression throughout the entire lockdown period. Future research should investigate a longitudinal comparison between media usage over time and COVID-19-related mental health issues.

## Limitations

The current study has several limitations. Data was collected cross-sectionally meaning it is reflective of a single point in time for participants. In turn, this means that variables are associated, and we cannot infer any directionality as we cannot infer cause and effect. Longitudinal data collection and analyses of variables over time would enable us to be more confident in any temporal relationships, for example, if depression/anxiety drives increased media consumption or if media consumption drives and increases depression/anxiety. Furthermore, given the self-reported nature of this data some participants may have provided answers that were subject to issues of recall bias. However, we feel this is a minimal concern given the saliency of the situation and the recency with which people were asked to report on their experiences (i.e. PHQ-9 and GAD-7 asks about experiences in the past 2 weeks). It is also pertinent to note that the survey assessed media usage only in regard to the frequency of checking social or traditional media and not by the average duration of each engagement with media. Future research should assess both frequency and duration of media usage for a more nuanced understanding of media usage. As with all socio/psychological research studies, there are many possible confounding variables that may or may not impact the relationships under investigation. We assessed several (but not all possible) group-based differences across key variables of interest and included only the confounding variables which were shown to be statistically significant; thus, we use only gender and age as our confounders.

Therefore, the study is limited as it is highly possible several pertinent confounding variables were still omitted from the analyses. It is also pertinent to note that the survey data is not representative of the UK population. The survey was administered as a rapid data collection exercise at the start of the UK’s first COVID-19 lockdown, and it was administered both via a participant data collection panel (PROLIFIC) and via social media channels. After finalising data collection, the PI of the project compared the collected data to the UK census and reported that males were underrepresented compared to males in the UK population (Armour et al. 2020). The resultant over-representation of females in the sample may have influenced the results as research has previously suggested that females have a higher morbidity rate than males regarding anxiety and depression (Matud 2017; Vlassoff 2007). Armour et al. (2020) further reported that employed individuals and students were oversampled; this is likely reflective of the nature of recruitment and data collection being entirely online; thus, those without access to devices and internet are unlikely to be represented in the data. Concerning ethnicity, these comparisons revealed that this was the variable which most closely represented the ethnic profiles across the UK nations (For a detailed discussion on data representativeness see Armour et al. 2020).

## Conclusion

In conclusion, this study indicated that higher media consumption was associated with higher levels of anxiety and depression. Worldwide it should be acknowledged that excessive media consumption, particularly social media relating to COVID-19, can have an effect on mental health. Education about how media platforms can be best utilised is important to consider. Harnessing media usage for peer support, exercise, telehealth and work may help overcome some psychological problems associated with COVID-19 media-related exposure. However, as this was a cross-sectional study, cause and effect cannot be determined; therefore, future research involving longitudinal data collection and analyses of variables is warranted.

## Data Availability

The data is not available to be shared publicly because of privacy and ethicalrestrictions. Dervived data supporting the findings of the study will be made available from thecorresponding author, and/or the PI (Armour: c.armour@qub.ac.uk) on reasonable request.

## Abbreviations

COVID-19: Coronavirus disease-19
GAD-7: Generalised Anxiety Disorder scale
PHQ: Patient Health Questionnaire
MDD: Major depressive disorder
